# Supplementary material for: Brain insulin sensitivity is linked to adiposity and body fat distribution
Source: Nat Commun. 2020 Apr 15;11:1841. doi: 10.1038/s41467-020-15686-y (PMC7160151; doi:10.1038/s41467-020-15686-y)
Supplement: Supplementary file 1 — Supplementary Information [file 41467_2020_15686_MOESM1_ESM.pdf]

Supplementary material for

Brain insulin sensitivity is linked to adiposity and body fat distribution

Stephanie Kullmann, Vera Valenta, Robert Wagner, Otto Tschritter, Jürgen Machann, Hans-Ulrich Häring, Hubert Preissl, Andreas Fritsche, and Martin Heni

Supplementary Tables

Supplementary Table 1: Participants´ characteristics of the longitudinal TULIP study

Characteristic	Baseline	at 24 months	at long-term follow-up
**N**	15		
**Sex (f/m)**	7/8		
**Age (y)**	45 ± 2	46 ± 2	54 ± 2
**BMI (kg/m^2)**	30.2 ± 0.8	29.7 ± 0.9	30.2 ± 0.8
**Total adipose tissue (l)**	33.2 ± 2.8	29.7 ± 2.8	34.7 ± 2.9
**Visceral adipose tissue (l)**	4.1 ± 0.5	3.5± 0.5	5.2 ± 0.8
**Subcutaneous adipose tissue (l)**	12.2 ± 0.9	10.6 ± 1.1	12.3 ± 1.0

Presented are means ± SEM.

Supplementary Table 2: Participants´ characteristics of the cross-sectional cohort

Characteristic	
**N**	112
**Sex (f/m)**	53/59
**Age (y)**	39 ± 2
**BMI (kg/m^2)**	28.4 ± 0.5
**Total adipose tissue (l)**	31.9 ± 1.3
**Visceral adipose tissue (l)**	3.4 ± 0.2
**Subcutaneous adipose tissue (l)**	12.6 ± 0.5

Presented are means ± SEM.

Supplementary Table 3: Changes in body composition and metabolism during and after 9 years of lifestyle intervention

	brain insulin resistant (N=8)		brain insulin sensitive (N=7)		pManova (brain insulin sensitivity x time)
	mean	SEM	mean	SEM	
**Change in body weight (kg)**					0.05
**after 9 months**	-0.94	1.37	-4.46	1.42	
**after 24 months**	1.44	1.32	-5.80	2.80	
**after 9 years**	1.63	1.39	-5.06	3.59	
**Change in BMI (kg/m^2)**					0.08
**after 9 months**	-0.37	0.49	-1.49	0.45	
**after 24 months**	0.55	0.47	-1.66	0.91	
**after 9 years**	0.94	0.44	-1.22	1.04	
**Change in WHR**					0.0478
**after 9 months**	0.00	0.02	-0.04	0.01	
**after 24 months**	-0.02	0.02	-0.05	0.01	
**after 9 years**	0.02	0.02	-0.02	0.03	
**Change in total adipose tissue (l)**					0.0128
**after 9 months**	-0.98	1.35	-5.90	2.04	
**after 24 months**	0.85	1.26	-7.99	2.28	
**after 9 years**	5.24	1.30	-2.72	4.38	
**Change in visceral adipose tissue (l)**					0.0092
**after 9 months**	-0.24	0.17	-0.86	0.30	
**after 24 months**	-0.03	0.26	-1.08	0.30	
**after 9 years**	1.18	0.35	0.29	0.65	
**Change in subcutaneous adipose tissue (l)**					0.07
**after 9 months**	-0,15	0,65	-1,97	0,74	
**after 24 months**	0,03	0,43	-3,39	0,79	
**after 9 years**	1,69	0,25	-0,65	1,22	

The group was stratified by the median brain response to insulin infusion, corrected for response to saline infusion, assessed by MEG as change in theta frequency band. P values are from MANOVAs with theta band response as a continuous variable.
