# Supplementary material for: Brain insulin sensitivity is linked to adiposity and body fat distribution
Source: Nat Commun. 2020 Apr 15;11:1841. doi: 10.1038/s41467-020-15686-y (PMC7160151; doi:10.1038/s41467-020-15686-y)
Supplement: Supplementary file 3 — Reporting Summary [file 41467_2020_15686_MOESM3_ESM.pdf]

nature research

Corresponding author(s): Martin Heni

Last updated by author(s): 22/01/2020

Reporting Summary

Nature Research wishes to improve the reproducibility of the work that we publish. This form provides structure for consistency and transparency in reporting. For further information on Nature Research policies, see Authors & Referees and the Editorial Policy Checklist .

Statistics

For all statistical analyses, confirm that the following items are present in the figure legend, table legend, main text, or Methods section.

n/a	Confirmed	
☐	☒	The exact sample size (*n*) for each experimental group/condition, given as a discrete number and unit of measurement
☐	☒	A statement on whether measurements were taken from distinct samples or whether the same sample was measured repeatedly
☐	☒	The statistical test(s) used AND whether they are one- or two-sided<br>*Only common tests should be described solely by name; describe more complex techniques in the Methods section.*
☐	☒	A description of all covariates tested
☒	☐	A description of any assumptions or corrections, such as tests of normality and adjustment for multiple comparisons
☐	☒	A full description of the statistical parameters including central tendency (e.g. means) or other basic estimates (e.g. regression coefficient) AND variation (e.g. standard deviation) or associated estimates of uncertainty (e.g. confidence intervals)
☒	☐	For null hypothesis testing, the test statistic (e.g. *F*, *t*, *r*) with confidence intervals, effect sizes, degrees of freedom and *P* value noted<br>*Give P values as exact values whenever suitable.*
☒	☐	For Bayesian analysis, information on the choice of priors and Markov chain Monte Carlo settings
☒	☐	For hierarchical and complex designs, identification of the appropriate level for tests and full reporting of outcomes
☒	☐	Estimates of effect sizes (e.g. Cohen's *d*, Pearson's *r*), indicating how they were calculated

Our web collection on statistics for biologists contains articles on many of the points above.

Software and code

Policy information about availability of computer code

Data collection	NA
Data analysis	JMP, Version 13 (SAS Institute, Cary, NC, USA).

For manuscripts utilizing custom algorithms or software that are central to the research but not yet described in published literature, software must be made available to editors/reviewers. We strongly encourage code deposition in a community repository (e.g. GitHub). See the Nature Research guidelines for submitting code & software for further information.

Data

Policy information about availability of data

All manuscripts must include a data availability statement. This statement should provide the following information, where applicable:

- Accession codes, unique identifiers, or web links for publicly available datasets
- A list of figures that have associated raw data
- A description of any restrictions on data availability

We will not be able to share patient level data due to local privacy protection regulations and due to the fact that participants did not consent to this.

Field-specific reporting

Please select the one below that is the best fit for your research. If you are not sure, read the appropriate sections before making your selection.

☒ Life sciences ☐ Behavioural & social sciences ☐ Ecological, evolutionary & environmental sciences

For a reference copy of the document with all sections, see nature.com/documents/nr-reporting-summary-flat.pdf

Life sciences study design

All studies must disclose on these points even when the disclosure is negative.

Sample size	There was no formal sample size determination for the MEG subgroup when the TULIP study started more than 10 years ago. In the longitudinal study 28 participants were included, 15 participants reached the follow up after nearly 10 years. All these participants are included in the current analyses.<br>For the "replication" fMRI cohort, all 112 subjects from our database with available data on both fMRI with nasal insulin and whole-body MRI were included in the analysis. Therefore, we did not perform a sample size calculation for this group.
Data exclusions	No data was excluded.
Replication	After our findings in the longitudinal MEG cohort, we investigated cross-sectional data with fMRI measurements of brain insulin sensitivity in a replication attempt. We chose this approach as we are not aware of any other cohort with determination of brain insulin sensitivity and follow-up whole-body MRI measurements.
Randomization	Not applicable as this is not a randomized study.
Blinding	At data collection, investigators were blinded to the brain insulin sensitivity of participants.

Reporting for specific materials, systems and methods

We require information from authors about some types of materials, experimental systems and methods used in many studies. Here, indicate whether each material, system or method listed is relevant to your study. If you are not sure if a list item applies to your research, read the appropriate section before selecting a response.

Materials & experimental systems

n/a	Involved in the study
☒	☐ Antibodies
☒	☐ Eukaryotic cell lines
☒	☐ Palaeontology
☒	☐ Animals and other organisms
☐	☒ Human research participants
☐	☒ Clinical data

Methods

n/a	Involved in the study
☒	☐ ChIP-seq
☒	☐ Flow cytometry
☐	☒ MRI-based neuroimaging

Human research participants

Policy information about studies involving human research participants

Population characteristics	This is presented as supplementary tables 1 and 2 of the manuscript.
Recruitment	Participants were recruted by advertisments in newspapers, University-wide email anouncements and leafelets.
Ethics oversight	The Ethics Committee of the University of Tübingen approved the studies.

Note that full information on the approval of the study protocol must also be provided in the manuscript.

Clinical data

Policy information about clinical studies

All manuscripts should comply with the ICMJEguidelines for publication of clinical research and a completedCONSORT checklist must be included with all submissions.

Clinical trial registration	MRI data are from NCT02991365, NCT01797601, NCT01847456, NCT02468999, NCT02870361, and NCT03227484
Study protocol	As this is pooled data from our database, there is not a single study protocol. However, we will share protocols of these studies upon request.
Data collection	All data was collected at the University Hospital Tübingen between 2003 and 2018.
Outcomes	*Describe how you pre-defined primary and secondary outcome measures and how you assessed these measures.*

Magnetic resonance imaging

Experimental design

Design type	resting state
Design specifications	two 5 minute resting-state cerebral blood flow measurements before and 30 min after intranasal insulin
Behavioral performance measures	NA

Acquisition

Imaging type(s)	functional
Field strength	3 Tesla
Sequence & imaging parameters	Pulsed arterial spin labeling (PASL) images were obtained with a PICORE-Q2TIPS. A total of 16 axial slices with a slice thickness of 5 mm (1.00-mm gap) were acquired in ascending order. Each measurement consisted of 79 alternating tag and control images with the following imaging parameters: inversion time (TI), TI1 = 700 ms, TI2 = 1800 ms, repetition time (TR) = 3000 ms, echo time (TE) = 19 ms, inplane resolution = 3x3 mm2, field of view = 192 mm, matrix size 64x 64 and flip angle = 90°.
Area of acquisition	Whole-brain

Diffusion MRI ☐ Used ☒ Not used

Preprocessing

Preprocessing software	Image preprocessing was performed by using the ASLtbx with SPM8. Functional images were coregistered to the individual anatomical image and smoothed (full width at half maximum: 6 mm).
Normalization	The high resolution T1-weighted image was normalized in Montreal Neurological Institute space (1 x 1 x 1 mm) using SPM8's unified segmentation normalization, and the resulting parameter file was used with the individual coregistered CBF maps in normalized space (3 x 3 x 3 mm).
Normalization template	normalized in Montreal Neurological Institute (MNI305)
Noise and artifact removal	Six head motion parameters. No participant had head motion with more than 2.0 mm maximum displacement or 2.0° of any angular motion.
Volume censoring	NA

Statistical modeling & inference

Model type and settings	NA
Effect(s) tested	NA

Specify type of analysis: ☐ Whole brain ☒ ROI-based ☐ Both

Anatomical location(s)	Hypothalamus based on recent publication (Kullmann et al 2015, Diab Care). Further statistics was performed in JMP.
Statistic type for inference (See Eklund et al. 2016)	NA
Correction	NA

Models & analysis

n/a	Involved in the study
☒	☐ Functional and/or effective connectivity
☒	☐ Graph analysis
☒	☐ Multivariate modeling or predictive analysis
